# Behavior of Male and Female C57BL/6J Mice Is More Consistent with Repeated Trials in the Elevated Zero Maze than in the Elevated Plus Maze

**DOI:** 10.3389/fnbeh.2017.00013

**Published:** 2017-01-26

**Authors:** Laura B. Tucker, Joseph T. McCabe

**Affiliations:** ^1^Pre-Clinical Studies Core, Center for Neuroscience and Regenerative MedicineBethesda, MD, USA; ^2^Department of Anatomy, Physiology and Genetics, F.E. Hébert School of Medicine, Uniformed Services University of the Health SciencesBethesda, MD, USA

**Keywords:** anxiety, memory, sex differences, behavior, one-trial tolerance

## Abstract

The elevated plus maze (EPM) and elevated zero maze (EZM) are behavioral tests that are widely employed to assess anxiety-like behaviors in rats and mice following experimental manipulations, or to test the effects of pharmacological agents. Both tests are based on approach/avoidance conflict, with rodents perceived as “less anxious” being more willing to explore the brighter, open and elevated regions of the apparatus as opposed to remaining in the darkened and enclosed regions. The goal of this research was to compare, under identical laboratory conditions, the behavior of male and female C57BL/6J mice in EZM and EPM during repeated trials. Mice were tested either daily or weekly, exclusively in the EPM or EZM, for a total of five exposures. During the first trial, the mazes were explored equally as measured by the total distance traveled during the test session. However, mice tested in the EZM spent nearly twice the amount of time in the anxiogenic regions (open quadrants) as the mice tested in the EPM spent in the open arms of that apparatus. After the first trial in the EPM, amounts of ambulation and percent time in the open arms decreased significantly (independent of inter-trial interval) which has been well-described in previous research as the one-trial tolerance phenomenon. In contrast, behavior in the EZM remained comparatively stable for several trials when the animals were tested weekly or daily. Sex differences were limited to activity levels, with females being more active than males. In conclusion, the design of the EZM encourages greater exploration of the anxiogenic regions of the apparatus, and may also be a more suitable test than the EPM for experimental designs in which assessment of anxiety-related behaviors is needed at more than one time point following experimental manipulations.

## Introduction

For decades, the elevated plus maze (EPM) has been the most popular test for the evaluation of anxiety-like states of rodents following experimental manipulations and for testing pharmacological agents (Griebel and Holmes, 2013; Haller et al., 2013). The test was developed and pharmacologically validated in rats (Pellow et al., 1985) and mice (Lister, 1987), and is comprised of two enclosed arms and two exposed arms joined by a central square. Shepherd et al. (1994) modified the design into an elevated “zero-maze” (EZM) with alternating “open” and “closed” quadrants, which had the benefit of removing the central square region of the EPM, resulting in simpler analysis of closed and open quadrants only. Interpretation of time spent in the central square of the EPM is often difficult, and was particularly a problem in mice as they can spend at least 20%–30% of the test session in this region (Lee and Rodgers, 1990; Rodgers et al., 1992). The EZM also eliminated the “boxed-in” regions of the closed arms of the EPM, allowing more continuous exploration of the apparatus.

The EPM and the EZM (as well as other tests including the open field and light-dark box) are referred to as unconditioned, “approach-avoidance” tasks that rely on rodents’ innate conflict between approaching and exploring (foraging) vs. avoiding potentially dangerous areas (Cryan and Holmes, 2005; Cryan and Sweeney, 2011). The bright and exposed regions of the EPM and EZM represent the “dangerous” areas of the mazes, whereas the darkened and enclosed regions are perceived as safer. Although significant criticisms of these simple tests should be noted, and on their own unconditioned tests of anxiety are insufficient for meeting the needs of all pre-clinical research in anxiety (Haller et al., 2013; Ennaceur and Chazot, 2016), they will likely remain in use due to their extensive history and ease of use, and are thus worth further exploration for understanding as models of anxiety-like behaviors in rodents.

Although the two tests are based on the same concept, designed and carried out similarly, and results are interpreted in the same way, it can be unclear which test is more appropriate to employ in a given experimental design as the output is not necessarily identical. The goal of this research was to compare, under identical testing conditions, the behavior of male and female C57BL/6J mice in the EPM and EZM. As behavior in the EPM is known to change with repeated trials, mice were tested at daily or weekly intervals for a total of five trials.

## Materials and Methods

### Animals

Male and cycling female C57BL/6J mice were obtained from Jackson Laboratories (Bar Harbor, ME, USA, Cat. No. 0664) and housed in facilities accredited by the Association for the Advancement and Accreditation of Laboratory Animal Care. Mice were group-housed (typically five in each cage) with standard enrichment (cotton nestlets and huts or igloos). Food (Harlan Teklad Global Diets 2018, 18% protein) and water were available *ad libitum* and the room was on a standard 12:12 h light:dark cycle. Animals were acclimated to housing facilities for at least 10 days prior to the beginning of the experiment, at which time they were approximately 9 weeks old. All procedures described were approved by the Institutional Animal Care and Use Committee at the Uniformed Services University of the Health Sciences (Bethesda, MD, USA).

### Behavioral Testing

**Figure 1:**
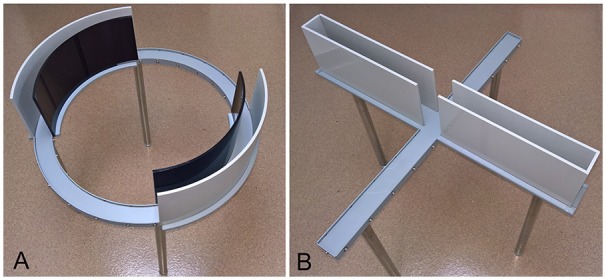
**The elevated zero maze (EZM; A)** and elevated plus maze (EPM; **B**). Both mazes are elevated 50 cm above the floor. The EZM **(A)** consists of four equal quadrants; two opposite quadrants are darkened and enclosed and the remaining two are open and exposed. The EPM **(B)** has two open, exposed arms; perpendicular to those arms are two darkened and enclosed arms. The four arms meet in a 5-cm square region. Illumination in both mazes is approximately 1600 Lux in the open regions and 200 Lux in the enclosed regions.

Behavioral testing apparatus are shown in Figure 1. Mice were randomly assigned to be tested exclusively on either the EZM or EPM, at either daily or weekly intervals for a total of five exposures to the apparatus. Experimental groups were represented in approximately equal numbers in several cohorts. The numbers of male and female mice tested in each maze and at each testing interval are shown in Table 1. EZM and EPM testing took place in the mornings and in the same room. The apparatus were obtained from Stoelting (Wood Dale, IL, USA) and both were elevated 50 cm above the floor. The EZM (Figure 1A) is an annular dark gray platform (60 cm in diameter) constructed of aluminum divided into four equal quadrants. Two opposite quadrants were “open”; the remaining two “closed” quadrants were surrounded by 16 cm high dark, opaque walls. Outer walls were constructed of dark gray plastic, inner walls were black Plexiglas. Quadrant lanes were 5 cm in width. The EPM (Figure 1B), also constructed of dark gray aluminum, consisted of two open arms (5 cm in width, 35 cm in length); perpendicular to the open arms were two closed arms of the same dimensions with opaque, dark gray plastic walls 16 cm high. The four arms met in a 5-cm center square region. Additional illumination for both mazes was provided by overhead fluorescent lamps; light levels in the open and closed regions of both mazes were approximately 1600 Lux and 200 Lux, respectively. The “open” regions of both mazes were surrounded by an edge approximately 1 cm high.

**Table 1:** **Number of male and female mice tested in each apparatus and testing interval**.

	Testing interval			
	Daily		Weekly	
	Male	Female	Male	Female
Elevated plus maze	25	25	20	20
Elevated zero maze	20	20	20	18

To start the EZM test, mice were placed at a randomly chosen boundary between an open and a closed zone, facing the inside of the closed zone. For EPM testing, mice were placed in the center of the maze, facing the inside of a closed arm. The tests were 5 min in duration. An overhead camera linked to a computer with Any-Maze software (Stoelting) tracked the position of the mouse and calculated the time spent in the open zones of the mazes, and the distance traveled during the test.

### Statistical Analyses

Statistical analyses were performed with SAS studio (SAS Institute Inc., Cary, NC, USA). A two-way analysis of variance (ANOVA; PROC GLM) was performed on all data collected on the first trial (combined for daily and weekly interval testing), with sex and apparatus as between-subjects factors. Data from each apparatus and testing interval were then tested separately with mixed model ANOVAs (PROC MIXED), with sex as a between-subjects factor and trial as a repeated measures factor. The Kenward-Roger degrees of freedom approximation and an autoregressive covariance structure (Lag-1) were employed for the repeated measures ANOVAs. Where significant main effects of day were found, Bonferroni-corrected *post hoc* tests (PROC PLM) compared results from day 1 to results from all subsequent days. Where significant day by sex interactions were found, Bonferroni-corrected planned contrasts (PROC PLM) were performed comparing males and females on each testing day. Figures were made using Microsoft Excel 2016 and Daniel’s XL Toolbox 6.60, and data in all figures represent the means ± standard error of the means.

## Results

### Comparison of EPM and EZM

**Figure 2:**
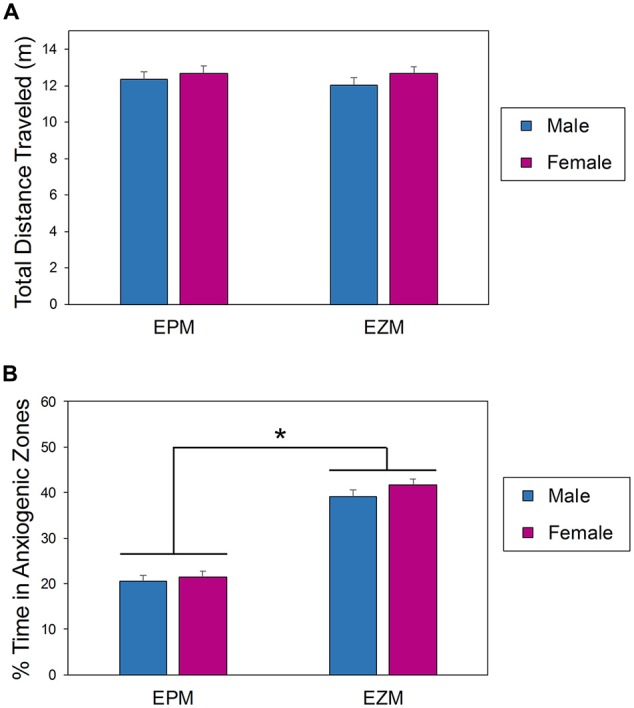
**Behavior of male and female mice in the EPM and EZM during the first exposure.** Amount of exploration (total distance traveled, **A**) was equal regardless of apparatus or sex. The amount of time spent in anxiogenic (bright and open) regions **(B)** was significantly dependent on apparatus but not sex. *EPM vs. EZM. EPM, elevated plus maze; EZM, elevated zero maze.

A two-way between subjects (sex × apparatus) ANOVA comparing activity levels of mice on the EPM and EZM during the first exposure to the apparatus found that there was no interaction effect between apparatus and sex (*F*_(1,164)_ = 0.148, *p* = 0.7005; Figure 2A). Mice were equally active regardless of sex (*F*_(1,164)_ = 1.360, *p* = 0.2452) or maze in which they were tested (*F*_(1,164)_ = 0.136, *p* = 0.7130; Figure 2A). There was a significant effect of apparatus (*F*_(1,164)_ = 202.92, *p* < 0.0001) on the percent of time spent in the open regions of the mazes (Figure 2B); the mice tested in the EZM spent significantly more time in the open quadrants of the maze than the mice tested in the EPM spent in the open arms (Figure 2B). Male and female mice behaved similarly in both mazes (*F*_(1,164)_ = 1.56, *p* = 0.2141), and there was no interaction between sex and apparatus (*F*_(1,164)_ = 0.34, *p* = 0.5606).

### Repeated Testing in the EPM

**Figure 3:**
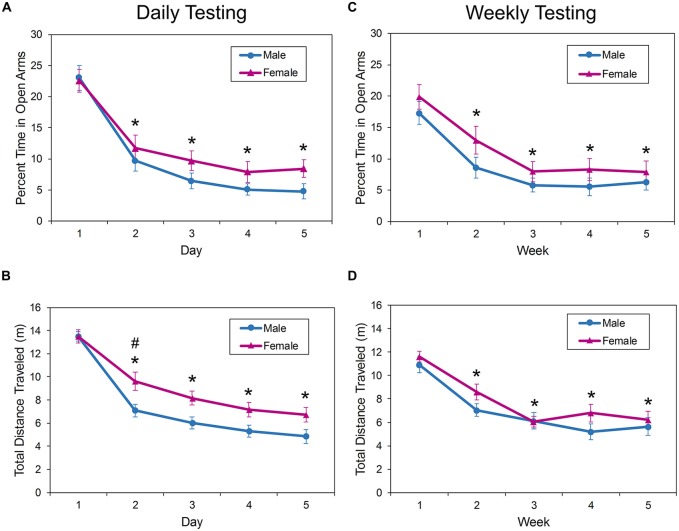
**Behavior of male and female mice in the EPM during repeated daily (A,B)** or weekly **(C,D)** testing. All mice habituated rapidly to the apparatus as evidenced by a significant decrease in exploration (distance traveled) after the first trial **(B,D)**. When the mice were tested daily, female mice ambulated greater distances on the second day of testing **(B)**. In addition, the amount of time spent exploring the open, anxiogenic regions of the EPM **(A,C)** were significantly decreased on all days following the first exposure. These changes did not depend on the testing interval. The asterisk (*) denotes a significant difference on the denoted day compared to Day 1. The pound sign (#) denotes a significant effect of sex on the indicated day. EPM, elevated plus maze; EZM, elevated zero maze.

Analysis of mice tested daily in the EPM showed a significant effect of trial on the percent of time spent in the open arms of the apparatus (*F*_(4,139)_ = 56.92, *p* < 0.0001; Figure 3A). Bonferroni-corrected *t*-tests showed that mice spent less time in the open arms on all trials after trial 1 (*p* < 0.0001), with the animals decreasing the percent of time in the open arms from about 22% on trial 1 to about 7% on trial 5. There was no effect of sex (*F*_(1,50.6)_ = 1.71, *p* = 0.1963) or sex by day interaction effect (*F*_(4,139)_ = 0.84, *p* = 0.4994) on time spent in the open arms.

There was a sex by day interaction effect on the amount of ambulation in the EPM with daily exposure (*F*_(4,114)_ = 2.79, *p* = 0.0297; Figure 3B). Bonferroni-adjusted planned contrasts performed for each testing day found that female mice were significantly more active in the EPM during the second exposure only (*p* = 0.0125). *Post hoc* comparisons of distance traveled on each day also found that animals were significantly more active in the EPM on day 1 than on all subsequent days (*p* < 0.0001).

Similar results were observed when the testing interval in the EPM was increased to 1 week (Figures 3C,D). There was a significant main effect of trial on the percent of time the mice spent in the open arms of the EPM (*F*_(4,108)_ = 34.29, *p* < 0.0001; Figure 3C); mice spent about 20% of the testing session in the open arms during the first trial, but the amount of time in the anxiogenic zones decreased to about 7% on the fifth trial.

The amount of ambulation as measured by the total distance traveled also decreased with increased exposure to the EPM at weekly intervals. There was a significant main effect of trial on distance traveled in the maze (*F*_(4,106)_ = 69.96, *p* < 0.0001; Figure 3D), but no interaction effect between sex and trial (*F*_(4,106)_ = 1.54, *p* = 0.1946) or main effect of sex (*F*_(1,38)_ = 1.28, *p* = 0.2658). The amount of maze exploration decreased between day 1 and all subsequent days (*p* < 0.0001).

### Repeated Testing in the EZM

**Figure 4:**
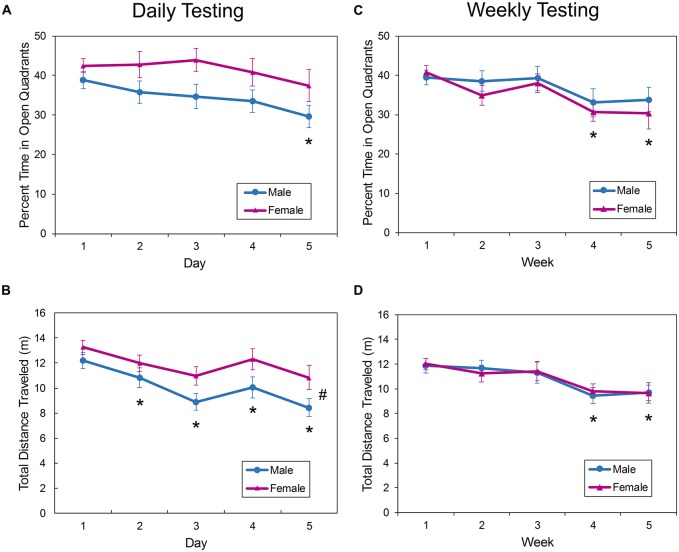
**Behavior of male and female mice in the EZM during repeated daily (A,B)** or weekly **(C,D)** testing. There was a main effect of sex on ambulation in the maze during daily testing, with female mice exploring the apparatus more than male mice **(B)**. With daily testing, the amount of exploration decreased after the first trial **(B)**, but this measure remained consistent until the fourth trial when the interval was increased to 1 week **(D)**. The time spent in the open quadrants also remained consistent with further exposure to the EZM, with the percent of time not decreasing until trial 4 or 5 **(A,C)**. The asterisk (*) denotes a significant difference on the denoted day compared to Day 1. The pound sign (#) denotes a main effect of sex. EPM, elevated plus maze; EZM, elevated zero maze.

In the EZM, the amount of time spent in the open quadrants in the mice tested daily (Figure 4A) was not affected by sex (*F*_(1,38.3)_ = 4.00, *p* = 0.0527), nor was there a sex by day interaction effect on this measure (*F*_(4,106)_ = 0.060, *p* = 0.6613). There was a significant main effect of day on open quadrant time (*F*_(4,106)_ = 3.61, *p* = 0.0084). Activity in the open quadrants remained consistent from trials 1 to 4 (Figure 4A), but time in the quadrants during trial 5 was significantly less than during trial 1 (*p* = 0.0007). Over the five trials, the amount of time in the open quadrants decreased from approximately 40% to about 33%.

There was a significant main effect of sex on distance traveled in the EZM with female mice exploring the apparatus more than male mice (*F*_(1,38.1)_ = 4.91, *p* = 0.0328; Figure 4B). There was also a main effect of day/trial on distance traveled; after day 1, mice ambulated less on all subsequent trials (*p* < 0.0117).

With weekly exposure to the EZM for 5 weeks, the amount of time in the open quadrants was not affected by sex (*F*_(1,36.2)_ = 0.35, *p* = 0.5589) and there was no sex by trial interaction effect (*F*_(4,102)_ = 0.65, *p* = 0.6251). There was a main effect of trial (*F*_(4,102)_ = 7.05, *p* < 0.0001). The time in the open quadrants was decreased during the trials on week 4 (*p* < 0.0001) and 5 (*p* = 0.0001) compared to week 1 (Figure 4C).

Ambulation in the EZM was not affected by sex in mice tested in the EZM for 5 weeks (Sex by week interaction: *F*_(4,106)_ = 0.16, *p* = 0.9567; Main effect of sex: *F*_(1,36)_ = 0.00, *p* = 0.9525), but there was a significant effect of trial on the distance traveled (*F*_(4,106)_ = 6.95, *p* < 0.0001; Figure 4D). Mice were equally active during the first three trials, but there was a decrease in activity on the fourth (*p* < 0.0001) and fifth (*p* < 0.0001) trials compared to the first trial.

## Discussion

In this direct comparison of performance of male and female mice in the EPM and EZM, we found that although mice were equally active in both mazes when presented as novel environments, the animals tested in the EZM spent significantly more time in the anxiogenic “open” regions of the maze than the mice that were tested in the EPM. There are few studies in which the EPM and the EZM are both employed in the same laboratory as assays of anxiety-like behaviors. However, Amani et al. (2013) also tested separate groups of mice in the EZM and EPM, and showed that mice tested in the EPM only spent about half the amount of time in the open arms of the apparatus as the mice tested in the EZM spent in the open quadrants, which is very comparable to our current results. Greater amounts of time in the open quadrants of the EZM compared to open arms of the EPM have also been observed when a within-subjects design was employed (Pearson et al., 2015). Braun et al. (2011) directly compared the two mazes in rats, and also reported increased times spent in anxiogenic regions of the EZM compared to the EPM.

The amount of exploration and the percent of time spent in the open arms of the EPM decreased significantly (by approximately 35% and 50%, respectively) in both male and female mice after the first trial in the apparatus regardless of whether they were tested daily or weekly. The “one-trial tolerance” phenomenon (OTTP) is well-documented in the EPM in both rats and mice, in which after a first exposure to the maze, rodents significantly decrease their exploration of the open arms on subsequent exposures, and anxiolytic agents (e.g., benzodiazepines) are no longer effective at increasing the amount of time spent in anxiogenic zones (e.g., File et al., 1990; Rodgers et al., 1992; Rodgers and Shepherd, 1993; Treit et al., 1993; Holmes and Rodgers, 1998, 1999; Zhou et al., 2015). There are extensive studies aimed at describing the conditions under which the OTTP occurs and the potential underlying mechanisms, discussion of which goes beyond the scope of this article (but see Holmes and Rodgers, 1999; Gomes and Nunes-De-Souza, 2009; Roy et al., 2009; Zhou et al., 2015). However, it is widely agreed that the OTTP makes the EPM an unsuitable assay for measuring anxiety-like traits in longitudinal studies.

It is possible that extending the inter-trial interval well beyond 1 week may allow re-testing in the EPM without carry-over effects from previous exposure to the maze. It has been shown that increasing the inter-trial interval to 28 days returns performance of saline-treated rats to that observed in trial 1 (Schneider et al., 2011), although a room change was also required to preserve the anxiolytic-like effects of midazolam during the second trial. Zhou et al. (2015) recently confirmed that a 28-day interval restores behavior to baseline performance in rats, but there are currently no studies that have attempted to extend the testing interval in mice to a degree that would eliminate the OTTP. However, many experimental designs may require sampling anxiety-related behaviors at shorter intervals, thus making the EPM a difficult test to employ for longitudinal studies.

In contrast to the EPM, behavior in the EZM remained relatively stable for several trials even when animals are tested daily; amounts of exploration and time spent in anxiogenic zones decreased much less than in the EPM over the duration of the experiment. Between the first and second trials, total amounts of exploration and the time spent in the open quadrants decreased by approximately 7% and 5%. Although the change in the amount of ambulation in the EZM from trial 1 to trial 2 was statistically significant during daily testing, the decrease was much smaller than that measured in the EPM (about 35%). When the testing interval in the EZM was extended 1 week, the amount of exploration remained consistent for three trials. Also unlike the EPM, the decrease in open quadrant activity between the first two trials was non-significant; these behaviors remained stable until at least the fourth trial. Behavior of rats in the EZM remains consistent when the testing interval is 1–2 months (Ajao et al., 2012; Kamper et al., 2013), and Blokland et al. (2012) concluded that reducing the testing interval to 24 h does not affect the stability of anxiolytic-like behavior of rats in the EZM for at least four trials. However, Cook et al. (2002) reported a significant decrease in the time spent in the open quadrants of the EZM when male C57BL/6J mice were tested 24 h following an initial trial. The percent time spent in the open quadrants in the first trial of that study was approximately 15% (compared to 40% in our study), the walls of the closed quadrants were transparent, and the test was performed in dim lighting (44 Lux). These large differences in baseline performance and testing conditions make direct comparisons difficult, and more studies are needed to determine if behaviors in the EZM can remain consistent over many trials as our data suggest, or if this assay, like the EPM, will also show habituation of behaviors over multiple trials in mice.

Significant sex differences in this study were limited to activity levels, with female mice ambulating greater distances in the mazes than male mice. We have previously reported increased activity levels in female mice compared to male mice in the EZM and in the open field test (Tucker et al., 2016a), and there is a long history of research largely concluding that female mice tend to be more active than their male counterparts (e.g., Archer, 1975; Kokras and Dalla, 2014), although not all studies support this conclusion (e.g., Bolivar et al., 2000; An et al., 2011). In the current study, there was a trend toward reduced anxiety-like behaviors in female mice (increased time in the open arms (EPM) and open quadrants (EZM)), and we previously found significantly increased time in the open quadrants of the EZM in female mice (Tucker et al., 2016a). Consistent with these results, higher levels of anxiety in male mice in the EPM have also been reported (Rodgers and Cole, 1993; Võikar et al., 2001; Gioiosa et al., 2007; Painsipp et al., 2007; Walf et al., 2008; Hendershott et al., 2016). Behavior of female mice in anxiety tests may be influenced by the estrus cycle (Galeeva and Tuohimaa, 2001; Gangitano et al., 2009; Walf et al., 2009; Koonce et al., 2012), and some studies have demonstrated an anxiolytic-like effect of ovarian hormones in mice (Frye et al., 2004; Olesen et al., 2011; Koonce and Frye, 2013). Despite these effects, the collective behavior of female mice in the EZM in the current study was just as consistent over multiple days as it was in males despite the estrus cycle. Furthermore, it has been demonstrated that the use of female mice at undetermined stages of the estrus cycle does not contribute to variability in the data of behavioral experiments (Prendergast et al., 2014; Tucker et al., 2016a,b).

It should be noted that dissociating anxiety from locomotion may be difficult in tests that rely on exploratory drive such as the EPM and EZM (O’Leary et al., 2013), and with sex differences in levels of activity, tests that rely less on exploration may have greater construct validity in pre-clinical studies of sex differences in anxiety (Kokras and Dalla, 2014). Furthermore, correlations between levels of activity and amount of time spent in anxiogenic regions of the EZM have been reported (Tarantino et al., 2000), lending further support to concerns that differences in anxiety-like behaviors may be confounded by variability in locomotor activity.

A limitation of this study is a lack of data on changes in the effects of anxiogenic and anxiolytic treatments over multiple trials in the EZM. The reduction in the efficacy of anxiolytic drugs in the EPM after a single trial is well-studied (the OTTP), but there is a paucity of data regarding this phenomenon, if it exists, in the EZM. In addition, future studies should look at changes in ethological or defensive behaviors such as stretch attenuated postures and head dips, as these are also parameters that are altered by repeated trials and pharmaceutical agents in the EPM, and considered important assessments in these mazes that help provide a more complete behavioral profile and are less influenced by levels of motor activity (Rodgers et al., 1997; Cryan and Holmes, 2005). Furthermore, differences in behaviors between mouse strains have been measured in both the EPM (e.g., Rodgers and Cole, 1993; Võikar et al., 2001; Rodgers et al., 2002; O’Leary et al., 2013) and the EZM (Tarantino et al., 2000; Cook et al., 2001; Milner and Crabbe, 2008), but direct comparisons of performance on both mazes in more strains are needed.

In conclusion, the design of the EZM encourages greater exploration of the open and brightened “anxiogenic” regions of the apparatus than that of the EPM, likely due to the absence of the central square and the requirement of passage through the open quadrants for continuous movement and exploration. Behaviors (levels of exploration and time spent in the open arms) significantly decreased after the first trial in the EPM (the OTTP); further studies are needed in mice to determine if extending the inter-trial interval well beyond 1 week will allow longitudinal testing with this apparatus. In contrast, in the EZM the same behaviors remained constant for at least three trials in our laboratory testing conditions (independent of inter-trial interval), suggesting this assay may be much more suitable for experimental designs that require the measurement of anxiety-related behaviors at multiple time points following experimental manipulations. However, the high baseline levels of exploration of anxiogenic regions of the EZM (in our laboratory conditions) may render it less sensitive than the EPM to anxiolytic agents; further pharmacological characterization of this apparatus is warranted.

## Author Contributions

LBT designed the experiment, collected and analyzed the data and wrote the manuscript. JTM served as principal investigator, contributing to the design of the experiment and analysis of the data, and assisted with the manuscript.

## Funding

This work was supported by The Center for Neuroscience and Regenerative Medicine, 60855-300600-7.01.

## Disclaimer

The opinions, interpretations, conclusions and recommendations are those of the authors and are not necessarily endorsed by the U.S. Army, Department of Defense, the U.S. Government or the Uniformed Services University of the Health Sciences. The use of trade names does not constitute an official endorsement or approval of the use of such commercial hardware or software. This document may not be cited for purposes of advertisement.

## Conflict of Interest Statement

The authors declare that the research was conducted in the absence of any commercial or financial relationships that could be construed as a potential conflict of interest.

## References

[B1] Ajao D. O. Pop V. Kamper J. E. Adami A. Rudobeck E. Huang L.. (2012). Traumatic brain injury in young rats leads to progressive behavioral deficits coincident with altered tissue properties in adulthood. J. Neurotrauma 29, 2060–2074. 10.1089/neu.2011.1883 22697253 PMC3408248

[B2] Amani M. Samadi H. Doosti M. H. Azarfarin M. Bakhtiari A. Majidi-Zolbanin N.. (2013). Neonatal NMDA receptor blockade alters anxiety- and depression-related behaviors in a sex-dependent manner in mice. Neuropharmacology 73, 87–97. 10.1016/j.neuropharm.2013.04.056 23688920

[B3] An X.-L. Zou J.-X. Wu R.-Y. Yang Y. Tai F.-D. Zeng S.-Y.. (2011). Strain and sex differences in anxiety-like and social behaviors in C57BL/6J and BALB/cJ mice. Exp. Anim. 60, 111–123. 10.1538/expanim.60.111 21512266

[B4] Archer J. (1975). Rodent sex differences in emotional and related behavior. Behav. Biol. 14, 451–479. 10.1016/s0091-6773(75)90636-7 1100044

[B5] Blokland A. Ten Oever S. van Gorp D. van Draanen M. Schmidt T. Nguyen E.. (2012). The use of a test battery assessing affective behavior in rats: order effects. Behav. Brain Res. 228, 16–21. 10.1016/j.bbr.2011.11.042 22173002

[B6] Bolivar V. J. Caldarone B. J. Reilly A. A. Flaherty L. (2000). Habituation of activity in an open field: a survey of inbred strains and F1 hybrids. Behav. Genet. 30, 285–293. 10.1023/A:1026545316455 11206083

[B7] Braun A. A. Skelton M. R. Vorhees C. V. Williams M. T. (2011). Comparison of the elevated plus and elevated zero mazes in treated and untreated male Sprague-Dawley rats: effects of anxiolytic and anxiogenic agents. Pharmacol. Biochem. Behav. 97, 406–415. 10.1016/j.pbb.2010.09.013 20869983 PMC3006066

[B8] Cook M. N. Crounse M. Flaherty L. (2002). Anxiety in the elevated zero-maze is augmented in mice after repeated daily exposure. Behav. Genet. 32, 113–118. 10.1023/A:1015249706579 12036108

[B9] Cook M. N. Williams R. W. Flaherty L. (2001). Anxiety-related behaviors in the elevated zero-maze are affected by genetic factors and retinal degeneration. Behav. Neurosci. 115, 468–476. 10.1037//0735-7044.115.2.468 11345971

[B10] Cryan J. F. Holmes A. (2005). The ascent of mouse: advances in modelling human depression and anxiety. Nat. Rev. Drug Discov. 4, 775–790. 10.1038/nrd1825 16138108

[B11] Cryan J. F. Sweeney F. F. (2011). The age of anxiety: role of animal models of anxiolytic action in drug discovery. Br. J. Pharmacol. 164, 1129–1161. 10.1111/j.1476-5381.2011.01362.x 21545412 PMC3229755

[B12] Ennaceur A. Chazot P. L. (2016). Preclinical animal anxiety research—flaws and prejudices. Pharmacol. Res. Perspect. 4:e00223. 10.1002/prp2.223 27069634 PMC4804324

[B13] File S. E. Mabbutt P. S. Hitchcott P. K. (1990). Characterisation of the phenomenon of “one-trial tolerance” to the anxiolytic effect of chlordiazepoxide in the elevated plus-maze. Psychopharmacology (Berl) 102, 98–101. 10.1007/bf02245751 1975449

[B14] Frye C. A. Walf A. A. Rhodes M. E. Harney J. P. (2004). Progesterone enhances motor, anxiolytic, analgesic, and antidepressive behavior of wild-type mice, but not those deficient in type 1 5 α-reductase. Brain Res. 1004, 116–124. 10.1016/j.brainres.2004.01.020 15033426

[B15] Galeeva A. Tuohimaa P. (2001). Analysis of mouse plus-maze behavior modulated by ovarian steroids. Behav. Brain Res. 119, 41–47. 10.1016/s0166-4328(00)00341-7 11164524

[B16] Gangitano D. Salas R. Teng Y. Perez E. De Biasi M. (2009). Progesterone modulation of α5 nAChR subunits influences anxiety-related behavior during estrus cycle. Genes Brain Behav. 8, 398–406. 10.1111/j.1601-183X.2009.00476.x 19220484 PMC2712346

[B17] Gioiosa L. Fissore E. Ghirardelli G. Parmigiani S. Palanza P. (2007). Developmental exposure to low-dose estrogenic endocrine disruptors alters sex differences in exploration and emotional responses in mice. Horm. Behav. 52, 307–316. 10.1016/j.yhbeh.2007.05.006 17568585

[B18] Gomes K. S. Nunes-De-Souza R. L. (2009). Implication of the 5-HT_2A_ and 5-HT_2C_ (but not 5HT_1A_) receptors located within the periaqueductal gray in the elevated plus-maze test-retest paradigm in mice. Prog. Neuropsychopharmacol. Biol. Psychiatry 33, 1261–1269. 10.1016/j.pnpbp.2009.07.015 19625008

[B19] Griebel G. Holmes A. (2013). 50 years of hurdles and hope in anxiolytic drug discovery. Nat. Rev. Drug Discov. 12, 667–687. 10.1038/nrd4075 23989795 PMC4176700

[B20] Haller J. Aliczki M. Gyimesine Pelczer K. (2013). Classical and novel approaches to the preclinical testing of anxiolytics: a critical evaluation. Neurosci. Biobehav. Rev. 37, 2318–2330. 10.1016/j.neubiorev.2012.09.001 22981935

[B21] Hendershott T. R. Cronin M. E. Langella S. McGuinness P. S. Basu A. C. (2016). Effects of environmental enrichment on anxiety-like behavior, sociability, sensory gating, and spatial learning in male and female C57BL/6J mice. Behav. Brain Res. 314, 215–225. 10.1016/j.bbr.2016.08.004 27498148

[B22] Holmes A. Rodgers R. J. (1998). Responses of Swiss-Webster mice to repeated plus-maze experience: further evidence for a qualitative shift in emotional state? Pharmacol. Biochem. Behav. 60, 473–488. 10.1016/s0091-3057(98)00008-2 9632231

[B23] Holmes A. Rodgers R. J. (1999). Influence of spatial and temporal manipulations on the anxiolytic efficacy of chlordiazepoxide in mice previously exposed to the elevated plus-maze. Neurosci. Biobehav. Rev. 23, 971–980. 10.1016/s0149-7634(99)00030-5 10580311

[B24] Kamper J. E. Pop V. Fukuda A. M. Ajao D. O. Hartman R. E. Badaut J. (2013). Juvenile traumatic brain injury evolves into a chronic brain disorder: behavioral and histological changes over 6months. Exp. Neurol. 250, 8–19. 10.1016/j.expneurol.2013.09.016 24076005 PMC3895624

[B25] Kokras N. Dalla C. (2014). Sex differences in animal models of psychiatric disorders. Br. J. Pharmacol. 171, 4595–4619. 10.1111/bph.12710 24697577 PMC4209934

[B26] Koonce C. J. Frye C. A. (2013). Progesterone facilitates exploration, affective and social behaviors among wildtype, but not 5α-reductase type 1 mutant, mice. Behav. Brain Res. 253, 232–239. 10.1016/j.bbr.2013.07.025 23886595 PMC3761366

[B27] Koonce C. J. Walf A. A. Frye C. A. (2012). Type 1 5α-reductase may be required for estrous cycle changes in affective behaviors of female mice. Behav. Brain Res. 226, 376–380. 10.1016/j.bbr.2011.09.028 21946309 PMC3381506

[B28] Lee C. Rodgers R. J. (1990). Antinociceptive effects of elevated plus-maze exposure: influence of opiate receptor manipulations. Psychopharmacology (Berl) 102, 507–513. 10.1007/bf02247133 1965750

[B29] Lister R. G. (1987). The use of a plus-maze to measure anxiety in the mouse. Psychopharmacology (Berl) 92, 180–185. 10.1007/bf00177912 3110839

[B30] Milner L. C. Crabbe J. C. (2008). Three murine anxiety models: results from multiple inbred strain comparisons. Genes Brain Behav. 7, 496–505. 10.1111/j.1601-183x.2007.00385.x 18182070

[B31] O’Leary T. P. Gunn R. K. Brown R. E. (2013). What are we measuring when we test strain differences in anxiety in mice? Behav. Genet. 43, 34–50. 10.1007/s10519-012-9572-8 23288504

[B32] Olesen K. M. Ismail N. Merchasin E. D. Blaustein J. D. (2011). Long-term alteration of anxiolytic effects of ovarian hormones in female mice by a peripubertal immune challenge. Horm. Behav. 60, 318–326. 10.1016/j.yhbeh.2011.06.005 21722643 PMC3166431

[B33] Painsipp E. Wultsch T. Shahbazian A. Edelsbrunner M. Kreissl M. C. Schirbel A.. (2007). Experimental gastritis in mice enhances anxiety in a gender-related manner. Neuroscience 150, 522–536. 10.1016/j.neuroscience.2007.09.024 17945426 PMC4359910

[B34] Pearson B. L. Defensor E. B. Blanchard D. C. Blanchard R. J. (2015). Applying the ethoexperimental approach to neurodevelopmental syndrome research reveals exaggerated defensive behavior in Mecp2 mutant mice. Physiol. Behav. 146, 98–104. 10.1016/j.physbeh.2015.03.035 26066729 PMC4584164

[B35] Pellow S. Chopin P. File S. E. Briley M. (1985). Validation of open:closed arm entries in an elevated plus-maze as a measure of anxiety in the rat. J. Neurosci. Methods 14, 149–167. 10.1016/0165-0270(85)90031-7 2864480

[B36] Prendergast B. J. Onishi K. G. Zucker I. (2014). Female mice liberated for inclusion in neuroscience and biomedical research. Neurosci. Biobehav. Rev. 40, 1–5. 10.1016/j.neubiorev.2014.01.001 24456941

[B37] Rodgers R. J. Cole J. C. (1993). Influence of social isolation, gender, strain, and prior novelty on plus-maze behaviour in mice. Physiol. Behav. 54, 729–736. 10.1016/0031-9384(93)90084-s 8248351

[B39] Rodgers R. J. Cao B.-J. Dalvi A. Holmes A. (1997). Animal models of anxiety: an ethological perspective. Braz. J. Med. Biol. Res. 30, 289–304. 10.1590/s0100-879x1997000300002 9246227

[B100] Rodgers R. J. Davies B. Shore R. (2002). Absence of anxiolytic response to chlordiazepoxide in two common background strains exposed to the elevated plus-maze: importance and implications of behavioural baseline. Genes Brain Behav. 1, 242–251. 10.1034/j.1601-183X.2002.10406.x 12882369

[B40] Rodgers R. J. Lee C. Shepherd J. K. (1992). Effects of diazepam on behavioural and antinociceptive responses to the elevated plus-maze in male mice depend upon treatment regimen and prior maze experience. Psychopharmacology (Berl) 106, 102–110. 10.1007/bf02253596 1738787

[B38] Rodgers R. J. Shepherd J. K. (1993). Influence of prior maze experience on behaviour and response to diazepam in the elevated plus-maze and light/dark tests of anxiety in mice. Psychopharmacology (Berl) 113, 237–242. 10.1007/bf02245704 7855188

[B41] Roy V. Chapillon P. Jeljeli M. Caston J. Belzung C. (2009). Free versus forced exposure to an elevated plus-maze: evidence for new behavioral interpretations during test and retest. Psychopharmacology (Berl) 203, 131–141. 10.1007/s00213-008-1378-2 18998112

[B42] Schneider P. Ho Y. J. Spanagel R. Pawlak C. R. (2011). A novel elevated plus-maze procedure to avoid the one-trial tolerance problem. Front. Behav. Neurosci. 5:43. 10.3389/fnbeh.2011.00043 21845176 PMC3146044

[B43] Shepherd J. Grewal S. Fletcher A. Bill D. Dourish C. (1994). Behavioural and pharmacological characterisation of the elevated “zero-maze” as an animal model of anxiety. Psychopharmacology (Berl) 116, 56–64. 10.1007/bf02244871 7862931

[B44] Tarantino L. M. Gould T. J. Druhan J. P. Bucan M. (2000). Behavior and mutagenesis screens: the importance of baseline analysis of inbred strains. Mamm. Genome 11, 555–564. 10.1007/s003350010107 10886023

[B45] Treit D. Menard J. Royan C. (1993). Anxiogenic stimuli in the elevated plus-maze. Pharmacol. Biochem. Behav. 44, 463–469. 10.1016/0091-3057(93)90492-c 8446680

[B46] Tucker L. B. Burke J. F. Fu A. H. McCabe J. T. (2016a). Neuropsychiatric symptom modeling in male and female C57BL/6J mice after experimental traumatic brain injury. J. Neurotrauma [Epub ahead of print]. 10.1089/neu.2016.4508 27149139 PMC5314988

[B47] Tucker L. B. Fu A. H. McCabe J. T. (2016b). Performance of male and female C57BL/6J mice on motor and cognitive tasks commonly used in pre-clinical traumatic brain injury research. J. Neurotrauma 33, 880–894. 10.1089/neu.2015.3977 25951234 PMC4860656

[B48] Võikar V. Kõks S. Vasar E. Rauvala H. (2001). Strain and gender differences in the behavior of mouse lines commonly used in transgenic studies. Physiol. Behav. 72, 271–281. 10.1016/s0031-9384(00)00405-4 11240006

[B50] Walf A. A. Koonce C. J. Frye C. A. (2008). Estradiol or diarylpropionitrile decrease anxiety-like behavior of wildtype, but not estrogen receptor beta knockout, mice. Behav. Neurosci. 122, 974–981. 10.1037/a0012749 18823154 PMC2562611

[B49] Walf A. A. Koonce C. Manley K. Frye C. A. (2009). Proestrous compared to diestrous wildtype, but not estrogen receptor beta knockout, mice have better performance in the spontaneous alternation and object recognition tasks and reduced anxiety-like behavior in the elevated plus and mirror maze. Behav. Brain Res. 196, 254–260. 10.1016/j.bbr.2008.09.016 18926853 PMC2614898

[B51] Zhou H. Yu C. L. Wang L. P. Yang Y. X. Mao R. R. Zhou Q. X.. (2015). NMDA and D_1_ receptors are involved in one-trial tolerance to the anxiolytic-like effects of diazepam in the elevated plus maze test in rats. Pharmacol. Biochem. Behav. 135, 40–45. 10.1016/j.pbb.2015.05.009 26004015

